# The Impact of Organizational Psychology Factors for the Cross-Border Legal Service Entrepreneurs

**DOI:** 10.3389/fpsyg.2020.01903

**Published:** 2020-08-11

**Authors:** Chengjin Xu, Zhe Zhang

**Affiliations:** ^1^School of Law, Shandong Normal University, Jinan, China; ^2^School of Economics and Management, Beijing Jiaotong University, Beijing, China

**Keywords:** trade friction, economic pressure, structural equation model, social support, the thought of entrepreneurial recession of entrepreneurs, value-chain potential

## Abstract

Trade friction has always been a prominent feature in the current economic development of the world. Its impacts on multinational enterprises are self-evident, but its psychological effects on the multinational entrepreneurs are still unclear. In order to understand the impacts of trade friction on psychological effects of multinational legal service entrepreneurs, 305 multinational entrepreneurs were selected in this study for questionnaire survey, and Spearman’s correlation and regression models were used to analyze the correlation among economic pressure, the thought of recession, self-efficacy, and social support. The structural equation model was used to analyze the influence path of economic pressure and social support on the thought of entrepreneurial recession, as well as the influence path of multinational entrepreneurship orientation and value-chain potential on the international performance. The results show that economic pressure is significantly and positively correlated with the thought of recession and self-efficacy extremely and significantly and negatively correlated with objective support and support utilization extremely; social support will reverse the thought of entrepreneurial recession caused by the economic pressure; the indirect impact path coefficient of social support utilization in economic pressure and entrepreneurial recession is – 0.281; the indirect impact path coefficient of value-chain potential in multinational entrepreneurial motivation and international performance is – 0.424. It shows that trade friction will indirectly trigger the thought of entrepreneurial recession of entrepreneurs by reducing their economic incomes. Besides, the social support utilization can significantly regulate the relationship between the economic pressure and the thought of entrepreneurial recession. Therefore, the value-chain potential plays an intermediary role in multinational entrepreneurial motivation and international performance.

## Introduction

In recent years, there is an upsurge of entrepreneurship in China. Nevertheless, there is a serious problem of oversupply in all fields; especially in the traditional fields, due to the increasing competitiveness in domestic market of China, it is difficult for more and more entrepreneurs to get success in the domestic market (Verbeke and Yuan, 2013). There are often many business opportunities in overseas markets, especially in developing countries. Therefore, Chinese entrepreneurs have shifted their entrepreneurial perspective to overseas markets in order to achieve success through overseas entrepreneurship. Multinational enterprise becomes one of the important forms of economic globalization, so it has been widely concerned by experts and scholars (Dimitratos et al., 2014; Da Silva Lopes et al., 2019). In order to enter the overseas market, multinational enterprises have made a lot of foreign investment, which can not only promote local economic development and employment but also benefit from technology spillovers (Tippmann et al., 2018). However, the work performance of multinational enterprises will also be affected by factors such as competition effects and government subsidy policies (Emelifeonwu and Valk, 2019). In addition, the entrepreneurship of multinational enterprises has to consider the profit transfer, fair trading, and other related points between the two countries. When there is a problem, there will be a “trade friction” (Jiang et al., 2019). China has now become the third largest trading country in the world, causing a shock on the original interest pattern, so the trade friction is inevitable. However, it is still unknown whether the trade frictions will affect the psychological effects of multinational entrepreneurs and in turn affect the work performance of these entrepreneurs.

With the deepening of economic globalization, the trend of entrepreneurial strategy and international diversification strategy are considered as key factors for multinational enterprises to succeed in the global market, and related studies have shown that the trend entrepreneurial strategy has a directly positive correlation with the entrepreneurial performance of multinational enterprises (McGee and Peterson, 2019; Palmer et al., 2019). The entrepreneur is the core of entrepreneurship, so the physical and psychological factors of entrepreneurs are critical, and the individual characteristics of entrepreneurs are the direct constraints of entrepreneurial activities in a specific entrepreneurial environment (Yueh et al., 2020). Studies have shown that the individual characteristics of entrepreneurs can also affect the entrepreneurial performance. Therefore, it is of great significance to explore the impacts of the trend of entrepreneurial strategy and the individual characteristics of entrepreneur on the work performance of multinational enterprises for enterprises to continue the multinational entrepreneurial activities and promote the development of enterprises (Dijkhuizen et al., 2018; Howell, 2018). Most of the entrepreneurial researches in the past had been dedicated to the outputs of small businesses and startups, instead of focusing on international acquisition scenarios.

In order to explore the impacts of the trend of internationalization on the psychological effects and work performance of multinational entrepreneurs, the legal service entrepreneurs of knowledge-based service companies were thus selected as the research objects. The impacts of trade friction on the psychological effects of multinational entrepreneurs and the impact mechanism of multinational entrepreneurial orientation on the international performance were explored in this study firstly. Finally, the validity and authenticity of hypotheses proposed in this study were verified through actual investigations. The results of this study aimed to lay a foundation for understanding the psychological effects of multinational entrepreneurs and promoting the international work performance of multinational enterprises.

## Literature Overview

### Psychological Effect of Entrepreneurs

Entrepreneurship can help entrepreneurs achieve their personal ideals and promote economic development and technological innovation. Researches in entrepreneurship-related fields had been conducted from the perspectives of management, psychology, and sociology. Lee et al. (2019) explored the differences between family and non-family senior management teams in organizational and psychological ownership of work, and the results showed that there was no significant difference between them and no significant impact on the entrepreneurship of the enterprise. Hooshangi and Loewenstein (2018) found that reduction in the investment opportunities could threaten the entrepreneur’s willingness to invest, and reduced investment costs would inhibit the investment decisions of pioneer entrepreneurs, while loose proprietary systems could have a positive impact on psychology of entrepreneurship. Zou et al. (2016) explored the different interests and goals of the inherent conflict between entrepreneurs and venture capitalists based on the psychological capital and then put forward a series of theoretical propositions about solving venture capital and strategic countermeasures. These studies revealed that the individual characteristics of entrepreneurs had a significant impact on entrepreneurial decision-making and entrepreneurship. Kautto (2019) found that the psychosocial impact of exogenous policy intervention could promote the transfer of entrepreneurship. Muhammad et al. (2020) indicated that external pressure had a positive and direct impact on behaviors of entrepreneurs and that the sustainable orientation of strategy can provide differentiation for enterprisers. In summary, psychological differences of individuals and external pressures affect the behaviors of entrepreneurs, yet the impacts of changes in the external environment and policies on the psychological effects of multinational entrepreneurs still need to be further explored.

### Impacts of International Performance of Multinational Entrepreneurs

Entrepreneurship is a significant form to achieve the social and economic development. In exploring the international entrepreneurship, it is very important to improve the performance of multinational enterprise. In order to explore the impact of strategic responsibilities of the multinational enterprises on the performance of their subsidiaries, Sarabi et al. (2020) found through building a model that the entrepreneurial leadership of the chief executive officer (CEO) of a subsidiary can improve the enterprise’s performance. Boone et al. (2019) revealed that the entrepreneurial spirit and the innovation enthusiasm of the top management team could affect the performance of the enterprise. In order to explore the impact of external crises on the performance of subsidiaries of the multinational enterprises, Martins et al. (2019) found that the sense of crisis affected the business performance of multinational enterprises through verification of the least-square structural equation model. Collings et al. (2019) revealed that the multinational strategy determined the goals of the global talent management system and also affected the performances of enterprises. From the perspective of individual human resources, it indicated that the personal performance could be improved by enlarging the human resources. Fernandes et al. (2018) constructed the framework of the multinational entrepreneur to affect the working performance, and the results showed that the entrepreneurial spirit can promote the working performance through management. Based on the above researches, it can be found that individual characteristics and strategic decisions of entrepreneurs can affect the work performance of multinational enterprises. However, the existing research lacks a definition of entrepreneurship-oriented dimension and its impact on the international performance of multinational companies.

To sum up, the multinational legal service entrepreneurs were taken as the research objects in this paper. In the form of questionnaire, the impact path of the economic pressure and social support of the trade friction on the entrepreneurial recession of entrepreneurs and the impact path of multinational entrepreneurial orientation and value-chain potential on the international performance of enterprises were analyzed. In this way, this study will lay the theoretical foundation for finding out the way to relieve from the psychological pressure of multinational entrepreneur and improving their working performance.

## Materials and Methods

### Research Objects

A total of 310 Chinese entrepreneurs of legal services who started businesses in the United States, Canada, Indonesia, Brazil, and the United Kingdom were selected as the research objects, the questionnaires were distributed online from June 2019 to December 2019, and 305 questionnaires were returned, so the response rate was 98.39%. The 305 subjects were 21–38 years old, with an average age of 26.88 ± 5.52. Studies show that the risk aversion of female entrepreneurs is higher than that of male and that self-efficiency has a strong predictive effect on the entrepreneurial behavior. Therefore, the gender, age, and self-efficiency were taken as the control variables for subsequent analysis.

### Model Building of Effect on Entrepreneur’s Recession From Economic Pressure and Social Support From the Trade Friction

**FIGURE 1:**
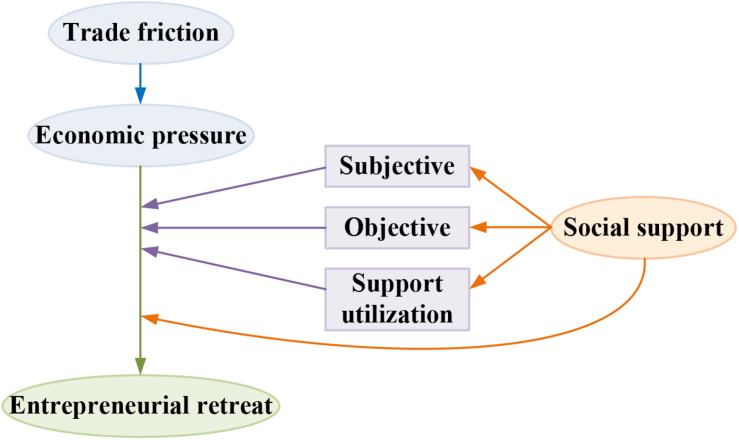
Model of effect of the entrepreneur’s thought of recession.

It is believed in this study that the economic pressure brought about by the trade friction could cause the entrepreneur to experience some negative emotions (anxiety, despair, depression, etc.). Based on the theory of learned helplessness, the theoretical model was built for effect of the economic pressure and social support caused by the trade friction on the entrepreneur’s thought of recession. The theoretical model is shown in Figure 1.

According to Figure 1, the economic pressure is an independent variable, entrepreneurial recession psychology is the dependent variable, and the sub-dimension under the social support is the intermediary variable. Therefore, the following hypotheses were proposed in this study:

a. The economic pressure could be promoted significantly by the trade friction;
b. The thought of entrepreneurial recession could be promoted significantly by the economic pressure;
c. The promotion of economic pressure on the thought of entrepreneurial recession could be regulated reversely by the social support;
c1. The promotion of economic pressure on the thought of entrepreneurial recession could be regulated reversely by the subjective support;
c2. The promotion of economic pressure on the thought of entrepreneurial recession could be regulated reversely by the objective support;
c3. The promotion of economic pressure on the thought of entrepreneurial recession could be regulated reversely by the support utilization.

### Model Building of Correlation on the Multinational Entrepreneurial Orientation and the International Performance

**FIGURE 2:**
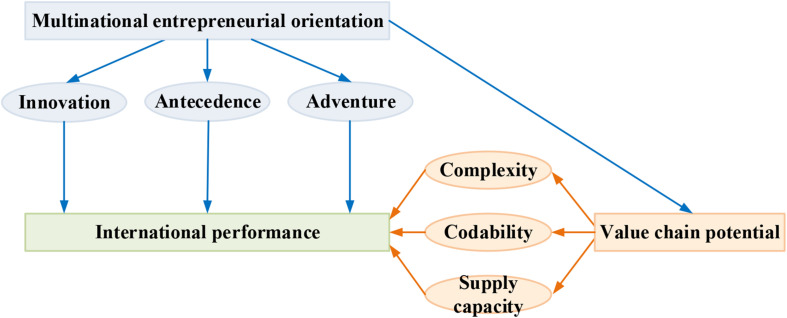
Model of effect of the entrepreneurship international performance.

In order to explore the correlation on the multinational entrepreneurial orientation and international performance, the theoretical model shown in Figure 2 below was proposed in this study by taking the global value chain as the intermediary variable.

Based on the above model, the below hypotheses are proposed in this study:

d. The international performance could be promoted by the multinational entrepreneurial orientation;
d1. The international performance could be promoted by innovation in the multinational entrepreneurial orientation;
d2. The international performance could be promoted by antecedence in the multinational entrepreneurial orientation;
d3. The international performance could be promoted by adventure in the multinational entrepreneurial orientation;
e. The international performance could be performed by the value-chain potential;
e1. The international performance could be performed by complexity of the trade;
e2. The international performance could be performed by codability of the product;
e3. The international performance could be performed by the supply capacity;
f. The value-chain potential is the intermediary variable for multinational entrepreneurial orientation to affect the international performance.

### Design of Evaluation Tools

The evaluation scales used in this study were all widely used, reliable, and valid evaluation tools in line with the actual conditions.

#### Evaluation Tools of the Social Support

The Social Support Rating Scale (SSRS) was used by Basinska and Rozkwitalska (2020). The scale included 10 items and 3 dimensions, which were the degree of subjective support, degree of objective support, and degree of support utilization. In addition, the Likert-4 rating method (1–4 scores indicate from “no” to “yes”) was used for evaluation in this scale. The subjective support included the support at the emotional level or resources from the relatives, friends, social networks, etc. The objective support refers to the actual support resources and also includes the material support provided by individuals, organizations, or the society. The support utilization refers to the utilization degree of the actual or perceived resource by individuals.

#### Evaluation Tools of the Economic Pressure

The Economic Pressure Rating Scale (EPRS) (Pollack et al., 2012) was compiled to evaluate the economic pressure of entrepreneur under the trade friction. The scale was supplemented by that “the recent economic environment has negatively affected my company,” “the business has been in trouble in the past year,” and “I have to bear a lot of pressures and responsibilities, and play a lot of roles.” The Likert-7 rating method (1–7 points indicate from “strongly disagree” to “strongly agree”) was used for evaluation in this scale.

#### Evaluation Tools of Entrepreneurial Recession

The exiting entrepreneurship assessment scale was used for recompilation and was applied to evaluate the entrepreneur’s thought of recession (Schuster et al., 2013). Three items were used for the evaluation, namely, “in the next 1 year, I will try my best to quit my current entrepreneurial activities,” “if I continue my business, I will be very worried,” and “continuing to start a business is not as passionate as when I started my business.” The Likert-7 rating method (1–7 points indicate from “strongly disagree” to “strongly agree”) was used for evaluation in this scale.

#### Evaluation Tools of Self-Efficiency

The entrepreneurship self-efficacy assessment scale (Chen et al., 2019) was used for recompilation and was applied to evaluate the entrepreneur’s self-efficacy. In this study, 17 items were used for the evaluation. The Likert-5 rating method (1–5 points indicate from “strongly disagree” to “strongly agree”) was used for evaluation in this scale.

#### Evaluation Tools of Multinational Entrepreneurial Orientation

Based on the three dimensions of innovativeness, proactiveness, and risk taking and the “9-item semantic difference scale” (Williams and Lee, 2009), the scale was improved by combining with the internationalization perspective of the multinational enterprises. The improved scale could be seen as below Table 1. When this scale was used for evaluation, it was indicated by “yes” for 1 score or “no” for 0 score of the evaluation.

**TABLE 1:** Evaluation scale of the multinational entrepreneurial orientation.

**Dimensionality**	**No.**	**Item**
Innovation	1	Focusing on marketing, products, or services? Or focusing on investment and leadership and innovation of the R & D and technology
	2	Having new products/services?
	3	Having great changes or the product/service path?
Proactiveness	4	Response to the competitor, or initiated by itself?
	5	The first to introduce the new product/service and the technology on management and operation?
	6	Avoiding competitive conflicts in the form of “peaceful coexistence” as often?
Risk-taking	7	Being inclined to the international project with low risk?
	8	Achieving the goals step by step in the international environment?
	9	Reducing the error rate of decision with an attitude of await-and-see?

#### Evaluation Tools of Value-Chain Potential

The value-chain potential was measured based on the trade complexity, product codability, and supply capacity (Rashid et al., 2019). The finally designed scale is shown in Table 2. When this scale was used for evaluation, it was indicated by “yes” for 1 score or “no” for 0 score of the evaluation.

**TABLE 2:** Evaluation scale of value-chain potential.

**Dimensionality**	**No.**	**Item**
Trade complexity	1	Having the need for information exchange by exchange, communication, and share
	2	High complexity for having the need for information exchange by exchange, communication, and share
Product codability	3	Quantity of information on related design or quality besides the formal documents
	4	High complexity for information on related design or quality besides the formal documents
Supply capacity	5	Strong acceptance to demand changes of the market compared with other competitors
	6	Strong acceptance to higher requirements from the service-provided side compared with other competitors
	7	High assets proprietary of the company
	8	High ability to bargain in the product trade

#### Evaluation Tools of International Performance

Based on the research results of other scholars (Abosede et al., 2018; Karami and Tang, 2019), the international performance was evaluated based on the four dimensions of foreign sales growth, changes in international market share, international investment return rate, and foreign operation satisfaction. The finally designed evaluation scale is shown in Table 3 below. When this scale was used for evaluation, it was indicated by “yes” for 1 score or “no” for 0 score of the evaluation.

**TABLE 3:** Evaluation scale of the international performance.

**No.**	**Item**
1	Rapid increase of the company’s overseas sales revenue in recent years
2	Rapider increase of the company’s sales revenue in the oversea markets than other major competitors
3	Rapid increase of overseas sales profits in recent years
4	Rapider increase of the company’s sales profits in the oversea markets than other major competitors
5	Rapid increase of the company’s overseas market shares in recent years
6	Rapider increase of the company’s overseas market shares than other major competitors
7	Higher return rate of the company’s oversea investment in recent years than other major competitors
8	Significant increase of the company’s sales rate in oversea market
9	High operational satisfaction of the company in oversea market in recent years
10	High satisfaction feedback of the company from the clients

### Statistical Analysis

The statistical processing was performed by SPSS 19.0. The validity tests of the evaluation tools were performed by using the Bartlett’s spherical test and KMO value (it was deemed as appropriation when KMO value was higher than 0.7, and it was deemed as appropriation when Bartlett’s spherical test *p* < 0.05). The reliability tests of the evaluation tools were performed by using the Cronbach’s α (it was deemed as high reliability when Cronbach’s α was higher than 0.7). The principal component analysis (PCA) was used to extract the factors, whose eigenvalue was higher than 1, in the variable tables. The descriptive statistics was used for statistical analysis of the sample distribution of each variable. The correlation of each variable was analyzed with the Pearson correlation. Hypotheses proposed in this study were verified by the process of hierarchical regression. When *P* < 0.05, it is considered that it has a statistical significance.

## Results

### Analysis on the Current Status of the Global and China-Related Trade Friction

Comparing the changes of the total number of the global and China-related trade frictions from 2009 to 2019, Figure 3 indicates that in the past 10 years, the trade frictions in the world have been more than 150 cases and those in China have been more than 70 cases. In 2019, the China-related trade frictions accounted for 30.46% of the cases in the whole world. Overall, the number of China-related trade friction showed a high trend all the time. The annual growth rate of the China-related trade friction showed the same trend as the annual growth rate of trade friction all over the world.

**FIGURE 3:**
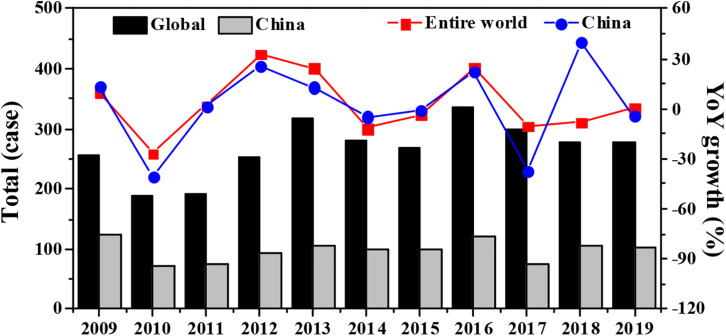
Change trends of total number of the global and China-related trade frictions from 2009 to 2019.

Comparing the proportions of the number of the global and China-related trade frictions from 2009 to 2019, Figure 4 shows that among all the global and China-related trade frictions, the anti-dumping cases accounted for the largest proportion, which was 80% (2363 cases) and 67% (715 cases), respectively, while the proportions of anti-subsidy were 12% (344 cases) and 14% (145 cases), respectively, and the proportions of safeguard precaution (8% (222 cases) and 9% (201 cases) had no great difference. In addition, there were also some trade frictions related to the special safeguards, accounting for 0% (10 cases) and 1% (10 cases), respectively. It shows that most countries in the world took the boycott measures such as increased taxation currently when dumping products from outside the country on the market. At the same time, the importing country offset the subsidies for imported products by imposing countervailing measures, price commitments, or import restrictions in order to protect the domestic industries and restore fair competition.

**FIGURE 4:**
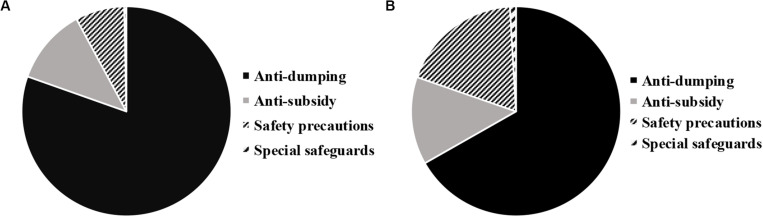
Proportions of the number of the global and China-related trade frictions from 2009 to 2019 [Figure **(A)** is the proportion of the global trade frictions; Figure **(B)** is the proportion of China-related trade frictions].

**FIGURE 5:**
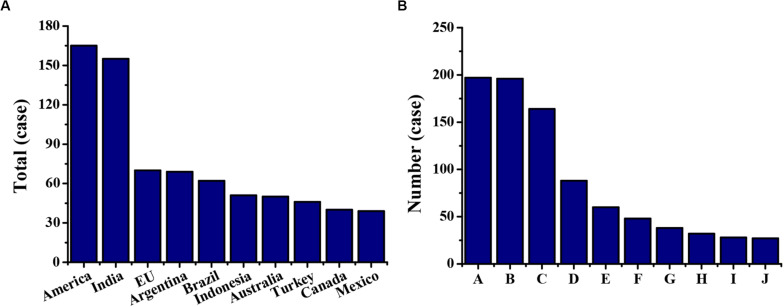
The top 10 complaining countries and the complained industries in the China-related trade frictions in 2009–2019. Figure **(A)** is the top 10 complaining countries; Figure **(B)** is the top 10 complained countries; A is for the fabricated metal industry; B is for the steel industry; C is for the chemical raw material and product industry; D is for the non-metallic product industry; E is for the textile industry; F is for the electrical industry; G is for the paper manufacturing industry; H is for the non-ferrous metal industry; I is for the food industry; and J is for the rubber product industry.

The top 10 complaining countries and the top 10 complained industries in the China-related trade frictions in 2009–2019 were compared. It could be seen from Figure 5A that, among the top 10 complaining countries, the cases both in the United States and in India were more than 150, and the number of complaints in the past 10 years was much higher than that in the European Union (EU) and other countries or organizations. It could be seen from Figure 5B that, among the top 10 complained industries, the numbers of metal product industry (197 cases), steel industry (196 cases), and chemical raw materials and product industries (164 cases) are much higher than those in other industries, and the most complained industries belong to the manufacturing industry.

### Descriptive Statistical Analysis on Basic Personal Information of the Entrepreneur

**FIGURE 6:**
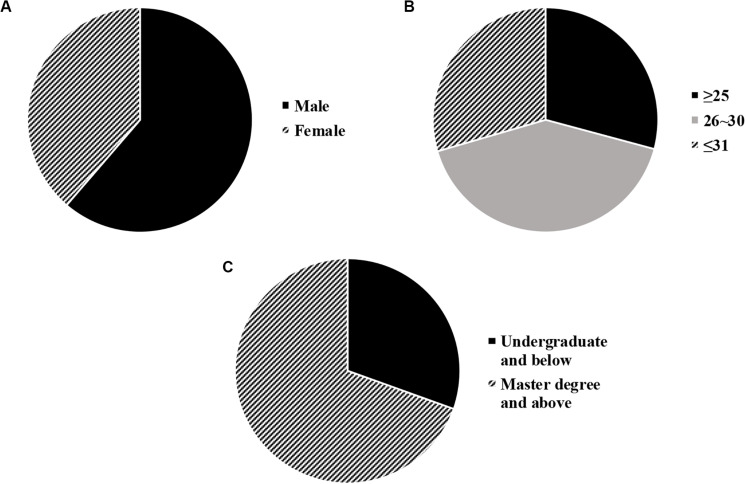
Statistical results of basic data of the subjects [Figure **(A)** is the sex ratio; Figure **(B)** is the age range; and Figure **(C)** is the academic qualification].

Then, a descriptive statistical analysis on the basic personal information of the entrepreneur was performed based on 305 entrepreneurs of legal services surveyed in this study. The results are shown in Figure 6 below. It could be seen that, among all the 305 entrepreneurs of legal services, the proportion in male is higher than that in female, which is 61% (187 cases) and 39% (118 cases), respectively, mainly aging range of 26–30 years with 126 cases (41%), and most are masters and above (212 cases, 70%).

### Results on Reliability and Validity Test of the Evaluation Tools

First, the reliability and validity test of the scales used in this study was performed. It could be seen from Table 4 that the Cronbach’s α is higher than 0.7, KMO is higher than 0.7, and *p* < 0.05 in the Bartlett spherical test. It shows that the evaluation tools used in this study have high reliability and validity, which lays the reliability of the subsequent evaluation results.

**TABLE 4:** Results on reliability and validity test of the evaluation tools.

**Scale**	**Cronbach’sα**	**KMO**	**Bartlett’s spherical test**		
			**χ ^2^**	***df***	***p***
Social support	0.815	0.713	720.955	44	0.000
Economic pressure	0.809	0.713	241.955	3	0.000
Entrepreneurial recession	0.821	0.711	225.782	3	0.000
Self-efficacy	0.952	0.712	267.893	3	0.000
Multinational entrepreneurial orientation	0.833	0.713	268.392	4	0.000
Value-chain potential	0.862	0.712	305.171	5	0.000
International performance	0.934	0.713	556.272	3	0.000

### Results of Correlation Analysis of Various Variables

Then, the correlation between the thought of entrepreneurial recession, the impact of entrepreneurial work performance, and other variables in this study were compared. The results are shown in Tables 5, 6 below. It could be seen from Table 4 that the economic pressure has an extremely significant positive correlation with the thought of recession and self-efficacy (*p* < 0.01) and has an extremely significant negative correlation with the objective support and support utilization (*p* < 0.01). The thought of recession has an extremely significant positive correlation with the self-efficacy (*p* < 0.01) and has a significant negative correlation with the objective support and support utilization (*p* < 0.05). The self-efficacy has a significant negative correlation with the subjective support and objective support (*p* < 0.05) and has an extremely significant negative correlation with the support utilization (*p* < 0.01). The subjective support has an extremely significant negative correlation with the objective support and support utilization (*p* < 0.01). There is an extremely significant positive correlation between the objective support and support utilization (*p* < 0.01).

**TABLE 5:** Correlation analysis on related variables of the thought of entrepreneurial recession.

**Variable**	**Economic pressure**	**Thought of recession**	**Self-efficacy**	**Subjective support**	**Objective support**	**Support utilization**
Economic pressure	1					
Thought of recession	0.432**	1				
Self-efficacy	0.321**	0.452**	1			
Subjective support	0.032	–0.101	−0.173*	1		
Objective support	−0.544**	−0.193*	−0.207*	−0.282**	1	
Support utilization	−0.501**	−0.704**	−0.686**	−0.457**	0.458**	1

*Correlation was significant at the 0.05 level (2-tailed); **Correlation was significant at the 0.01 level (2-tailed).

**TABLE 6:** Correlation analysis of related variables of the entrepreneurial working performance.

**Variable**	**Multinational entrepreneurial orientation**	**Innovation**	**Proactiveness**	**Risk-bearing**	**Value-chain potential**	**Transaction complexity**	**Product codability**	**Supply capacity**	**International performance**
Multinational entrepreneurial orientation	1								
Innovation	0.820**	1							
Proactiveness	0.810**	0.545**	1						
Risk-bearing	0.808**	0.467**	0.455**	1					
Value-chain potential	0.520**	0.460**	0.502**	0.348**	1				
Transaction complexity	0.398**	0.387**	0.362**	0.298**	0.832**	1			
Product codability	0.326**	0.178*	0.332**	0.274**	0.577**	0.202**	1		
Supply capacity	0.487**	0.434**	0.454**	0.303**	0.862**	0.622**	0.274**	1	
International performance	0.566**	0.551**	0.508**	0.347**	0.678**	0.567**	0.263**	0.656**	1

*Correlation was significant at the 0.05 level (2-tailed); **Correlation was significant at the 0.01 level (2-tailed).

It could be seen from Table 6 that the multinational entrepreneurial guide and its sub-dimensions (innovation, proactiveness, and risk-taking), value-chain potential, and its sub-dimensions (transaction complexity, product codability, and supply capacity) have a significant correlation with the international performance (*p* < 0.05).

### Verification Results of the Proposed Hypotheses

The hypotheses proposed earlier in this study were analyzed by the regression analysis. It could be seen from Table 7 that: a. The trade friction has an extremely significant positive impact on the economic pressure (β = 0.206; *p* < 0.01). b. The economic pressure has an extremely significant positive effect on the thought of entrepreneurial recession (β = 0.244; *p* < 0.01). c. The social support × economic pressure can negatively affect the thought of entrepreneurial recession, that is, the social support can regulate the thought of entrepreneurial recession caused by the economic pressure reversely (β = −0.192; *p* < 0.01). c1. and c2. The subjective support and objective support cannot regulate the thought of entrepreneurial recession caused by the economic pressure significantly (β = −0.078, −0.032; *p* > 0.05). c3. The support utilization can regulate the thought of entrepreneurial recession caused by the economic pressure (β = −0.281; *p* < 0.01). d. The multinational entrepreneurial orientation has a significant positive impact on the international performance (β = 0.532; *p* < 0.01). d1. The innovation dimension in multinational entrepreneurship has an extremely significant positive impact on the international performance (β = 0.288; *p* < 0.01). d2. The proactiveness dimension in multinational entrepreneurship has a significant positive impact on the international performance (β = 0.227; *p* < 0.01). d3. The risk-taking in the multinational entrepreneurship cannot affect the international performance (β = 0.036; *p* > 0.05). e. The value-chain potential has an extremely significant positive impact on the international performance (β = 0.798; *p* < 0.01). e1. The transaction complexity has an extremely significant positive impact on the international performance (β = 0.182; *p* < 0.01). e2. The product codability cannot affect the international performance (β = −0.092; *p* > 0.05). e3. The supply capacity has an extremely significant positive effect on the international performance (β = 0.424; *p* < 0.01). In addition, it is also found in this study that the multinational entrepreneurial orientation can affect the value-chain potential significantly (β = 0.415; *p* < 0.01).

**TABLE 7:** Analysis results of regression analysis models.

**Model**	**Independent variable**	**Dependent variable**	**β**	**△*R*^2^**	***p***
1	Trade friction	Economic pressure	0.206	0.344	0.000
2	Economic pressure	Thought of entrepreneurial recession	0.244	0.022	0.000
3	Economic pressure × social support	Thought of entrepreneurial recession	–0.192	0.021	0.000
4	Economic pressure × subjective support	Thought of entrepreneurial recession	–0.078	0.003	0.173
5	Economic pressure × objective support	Thought of entrepreneurial recession	–0.032	0.003	0.532
6	Economic pressure × support utilization	Thought of entrepreneurial recession	–0.281	0.032	0.000
7	Multinational entrepreneurial orientation	International performance	0.523	0.307	0.000
8	Innovation	International performance	0.288	0.322	0.000
9	proactiveness	International performance	0.227	0.314	0.000
10	Risk-bearing	International performance	0.036	0.004	0.102
11	Value-chain potential	International performance	0.798	0.443	0.000
12	Trade complexity	International performance	0.182	0.318	0.000
13	Product codability	International performance	–0.092	0.003	0.098
14	Supply capacity	International performance	0.424	0.261	0.000
15	Multinational entrepreneurial orientation	Value-chain potential	0.415	0.257	0.000

**× indicates that the social support and its sub-dimension exerts the intermediary role for impact of economic pressure on the thought of entrepreneurial recession.**

It could be seen from Table 8 that all the hypotheses are true except c1., C2., d3., and e2. Therefore, the result graph of the impact path proposed in this study has to be redrawn based on the verification results of the hypotheses.

**TABLE 8:** Verification results of the hypotheses.

**No.**	**Hypotheses**	**Verification result**
a.	The economic pressure could be promoted significantly by the trade friction.	Support
b.	The thought of entrepreneurial recession could be promoted significantly by the economic pressure.	Support
c.	The promotion of economic pressure on the thought of entrepreneurial recession could be regulated reversely by the social support.	Support
c1.	The promotion of economic pressure on the thought of entrepreneurial recession could be regulated reversely by the subjective support.	Not support
c2.	The promotion of economic pressure on the thought of entrepreneurial recession could be regulated reversely by the objective support.	Note support
c3.	The promotion of economic pressure on the thought entrepreneurial recession could be regulated reversely by the support utilization.	Support
d.	The international performance could be promoted by the multinational entrepreneurial orientation.	Support
d1.	The international performance could be promoted by innovation in the multinational entrepreneurial orientation.	Support
d2.	The international performance could be promoted by antecedence in the multinational entrepreneurial orientation.	Support
d3.	The international performance could be promoted by adventure in the multinational entrepreneurial orientation.	Not support
e.	The international performance could be performed by the value-chain potential.	Support
e1.	The international performance could be performed by complexity of the trade.	Support
e2.	The international performance could be performed by codability of the product.	Not support
e3.	The international performance could be performed by the supply capacity.	Support
f	The value-chain potential is the intermediary variable for multinational entrepreneurial orientation to affect the international performance.	Support

**FIGURE 7:**
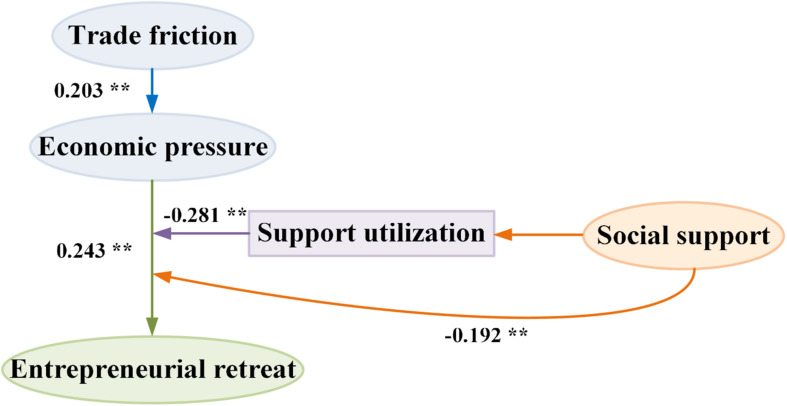
Impact paths of the economic pressure and social support based on the trade friction on the thought of entrepreneurial recession. ** represents *P* < 0.01.

The impact path of the regulated economic pressure and social support based on the trade friction on the thought of entrepreneurial recession is shown in Figure 7 below. It can be seen that the economic pressure could be impacted positively by the trade friction, while the occurrence of the thought of entrepreneurial recession could be impacted positively by the economic pressure. However, the thought of entrepreneurial recession caused by the economic pressure could be regulated by the social support and its support utilization.

**FIGURE 8:**
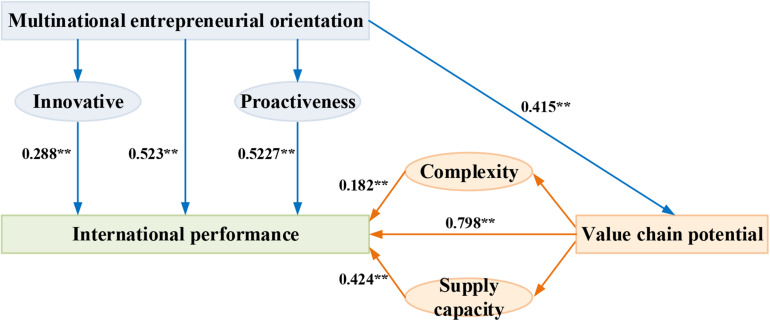
Impact path of the multinational entrepreneurial orientation, value-chain potential, and international performance. ** represents *P* < 0.01.

The impact paths of the regulated multinational entrepreneurial motivation, value-chain potential, and international performance are shown in Figure 8 below. It can be seen that the international performance could be promoted by the multinational entrepreneurial motivation and its innovation and proactiveness, and the international performance could be promoted by the transaction complexity and supply capacity of the value-chain potential. The value-chain potential can play a role of intermediation in the relationship between the multinational entrepreneurial motivation and international performance.

## Discussion

In this study, it is found that the United States, European Union, Association of Southeast Asian Nations, and Canada are main exporters of China, so the current export markets of China will continue to be the “main battlefield” of the trade friction in the future (Lawrence, 2018). On the other hand, exports from China to the emerging markets such as Russia, India, and Mexico also grow at 25% year by year, so the trade relations with the emerging markets will also be in the form of “no major problems and constant minor issues.” It is found that greater economic pressure to persons of multinational legal service can be promoted by the trade friction. Some early statistics in WTO show that 1 of 7 trade frictions is China-related (Holladay et al., 2017). In addition, after the trade frictions were analyzed in the whole world in the past 10 years, it is found that most trade frictions were China-related, and the China-related trade friction in 2019 only was more than 30%. With gradual development of the foreign trade, the trade friction has extended from a single product to the entire industry. Therefore, in the future, it should not only consider quelling trade disputes but also consider policy formulation and policy level of the trade friction (Ji et al., 2020).

The protection policies for the knowledge-based enterprises in various countries are not less than those for the manufacturing industry, and the incidents caused by trade friction for the knowledge-service enterprises entering their own countries are possible. Therefore, the legal service entrepreneurs have to bear greater economic pressure, so the trade friction may cause certain economic pressure on multinational entrepreneurs (Cui et al., 2019; Liu et al., 2019). When there are events such as trade friction that are beyond the scope of the entrepreneur’s own tolerance/control, they will have a helpless feeling. This negative emotion will greatly affect the entrepreneur’s pursuit of a sense of accomplishment, so excessive economic pressure will result in the psychological effect of entrepreneurial recession (Ryff, 2019). In the process of multinational entrepreneurship, even if there are a lot of subjective or objective social supports in the society, the entrepreneur fails to apply these supports in practice, then it will have no great significance for the economic pressure and psychological pressure of the entrepreneur. Thus, the support utilization in social support can regulate the thought of entrepreneurial recession caused by the economic pressure (Klyver et al., 2017; Monica et al., 2018). It indicated that entrepreneurs can improve the economic pressure caused by trade frictions in enterprises through reasonable utilization of the social support during the multinational entrepreneurship and then adjust their psychological effects. In the global market, the innovation of products, services, and management is the source of enterprise value. The advanced behavior of enterprise will bring first-mover advantages, which is conducive to seize the market share and thus obtain higher profits. Therefore, the innovation and advancement in the multinational entrepreneurial orientation can promote the international performance of enterprises (Álvarez Torres et al., 2019; Guzmán et al., 2019). In the process of product transactions, the multinational companies are committed to the development and improvement of services and other technologies, increase the complexity of market information in the transaction process, and improve the knowledge and other aspects. In this way, it can increase the initiative of the enterprise and satisfy the needs of customers to a greater extent. Therefore, the complexity of product transactions and supply capacity of product in the value-chain potential of entrepreneurship of the multinational enterprises can promote the international performance of enterprises (Bos et al., 2017; Ferreira et al., 2018; Biberhofer et al., 2019).

It is found that the entrepreneur’s thought of entrepreneurial recession can be affected positively by the size of economic pressure, which is similar to the results of Morris et al. (2020). Then, the regulation role of the social support and its sub-dimensions of subjective support, objective support, and support utilization to the thought of recession caused by the economic pressure was explored. The results of this study show that the stronger the social support, the weaker the relationship between thought of entrepreneurial recession caused by the economic pressure. The reasons for the above results may be that the research objects of this study are the multinational entrepreneurs. No matter how much subjective or objective support it has in the society, such support has to be applied in practice to improve the economic pressure of the enterprise and then reduce the thought of entrepreneurial recession (Alby et al., 2019), which is consistent with the results in this study that the subjective support and objective support cannot regulate the impact of economic pressure on the thought of entrepreneurial recession reversely. It is found in this study that the international performance could be promoted significantly by the multinational entrepreneurial orientation, and the effects of innovation and proactiveness in the multinational entrepreneurial orientation are significant. It is consistent with the results of Augustie and Saad (2019). The international performance cannot be impacted significantly by the risk-taking in the multinational entrepreneurial orientation. This may be because this experience in overseas operations of entrepreneurs is relatively weak in the international market, and the company’s management level is relatively insufficient, so that many problems behind the value-chain potential cannot be treated and solved skillfully and quickly. The value-chain potential is a key factor to drive the international performance (Vonsée et al., 2019). It is found in this study that the international performance can be promoted by the value-chain potential and its transaction complexity and supply capacity. This is because, in the transaction process, the enterprise is committed to the development and improvement of the services and other technologies, enhancement on complexity of the market information, and improvement of the knowledge. In this way, the initiative of the enterprise can be increased, and thus the clients can be satisfied to a larger extend. Entrepreneurship is a very complex and dynamic activity, and the difficulties experienced by multinational entrepreneurs are more than those experienced by domestic entrepreneurs (Boone et al., 2019). Regardless of any entrepreneurial behavior or activity, it is germinated in a specific entrepreneurial environment and developed step by step. Therefore, the entrepreneurial activities are inevitably affected by the entrepreneurial environment (Biryukov et al., 2020). In addition, it is found based on the codability of the products in this study that the codability of products can suppress the falseness of international performance. It is because codability of the products can facilitate the product or service management provided by the company in multinational enterprises, so that it is convenient to estimate and choose. Therefore, this variable has no inhibitory effect on the international performance of multinational enterprises.

## Conclusion

With assistance of statistical methods combined with questionnaire surveys, regression model, and structural equation models, this study analyzed the impact mechanism of trade friction on the mentality of multinational entrepreneurs. It was found that trade friction can trigger the thought of entrepreneurial recession of entrepreneurs indirectly by reducing their income. The social support utilization can significantly regulate the relationship between economic pressure and the thought of entrepreneurial recession. The multinational entrepreneurship motivation can promote the international performance of multinational enterprises through the value-chain potential. It fails to consider other factors of the external environment (such as policies, etc.) and the interactions from the personal internal factors to the thought of entrepreneurial recession and the entrepreneurial working performance. Accordingly, the quantity of samples has to be increased in the future and to take specific research in a certain area to explore the effects on external environment and the internal factors of individuals on the thought of entrepreneurial recession and entrepreneurial working performance. In conclusion, the thought of entrepreneurial recession of the entrepreneur can be reduced and the subjective initiative of the entrepreneur can be played by using the social support to the maximal extent. International performance of the enterprise can be improved by complying with the international market and improving the service ability. Results in this study can provide a basis for understanding the impact mechanism of the thought of entrepreneurial recession of entrepreneur of multinational legal service and its work performance.

## Data Availability Statement

The raw data supporting the conclusions of this article will be made available by the authors, without undue reservation, to any qualified researcher.

## Ethics Statement

The studies involving human participants were reviewed and approved by the Shandong Normal University Ethics Committee. The patients/participants provided their written informed consent to participate in this study. Written informed consent was obtained from the individual(s) for the publication of any potentially identifiable images or data included in this article.

## Author Contributions

CX wrote the manuscript. ZZ reviewed the manuscript. Both authors contributed to the article and approved the submitted version.

## Conflict of Interest

The authors declare that the research was conducted in the absence of any commercial or financial relationships that could be construed as a potential conflict of interest.
